# Brain computer interface learning for systems based on electrocorticography and intracortical microelectrode arrays

**DOI:** 10.3389/fnint.2015.00040

**Published:** 2015-06-10

**Authors:** Shivayogi V. Hiremath, Weidong Chen, Wei Wang, Stephen Foldes, Ying Yang, Elizabeth C. Tyler-Kabara, Jennifer L. Collinger, Michael L. Boninger

**Affiliations:** ^1^Department of Physical Medicine and Rehabilitation, University of PittsburghPittsburgh, PA, USA; ^2^Department of Veterans Affairs, Human Engineering Research LaboratoriesPittsburgh, PA, USA; ^3^Qiushi Academy for Advanced Studies (QAAS), Zhejiang UniversityHangzhou, China; ^4^Department of Bioengineering, University of PittsburghPittsburgh, PA, USA; ^5^Clinical and Translational Science Institute, University of PittsburghPittsburgh, PA, USA; ^6^Center for the Neural Basis of Cognition, Carnegie Mellon University and the University of PittsburghPittsburgh, PA, USA; ^7^Department of Neurological Surgery, University of PittsburghPittsburgh, PA, USA

**Keywords:** BCI learning, BCI mapping, brain control, human-computer interfaces, motor learning, cognitive skill learning

## Abstract

A brain-computer interface (BCI) system transforms neural activity into control signals for external devices in real time. A BCI user needs to learn to generate specific cortical activity patterns to control external devices effectively. We call this process BCI learning, and it often requires significant effort and time. Therefore, it is important to study this process and develop novel and efficient approaches to accelerate BCI learning. This article reviews major approaches that have been used for BCI learning, including computer-assisted learning, co-adaptive learning, operant conditioning, and sensory feedback. We focus on BCIs based on electrocorticography and intracortical microelectrode arrays for restoring motor function. This article also explores the possibility of brain modulation techniques in promoting BCI learning, such as electrical cortical stimulation, transcranial magnetic stimulation, and optogenetics. Furthermore, as proposed by recent BCI studies, we suggest that BCI learning is in many ways analogous to motor and cognitive skill learning, and therefore skill learning should be a useful metaphor to model BCI learning.

## Introduction

Brain-computer interface (BCI) technology aims to establish a direct communication pathway between the brain and external devices. BCI technology has the potential to assist, augment, or repair human sensorimotor and other cognitive functions, thus improving the quality of life for individuals with disabilities (Schwartz et al., 2006; Daly and Wolpaw, 2008; Donoghue, 2008; Moran, 2010; Wang et al., 2010c). During the last few decades, significant progress has been made in the development of BCI systems using various neural recording modalities, such as intracortical microelectrode arrays that record single/multi-unit activity (Taylor et al., 2002; Carmena et al., 2003; Hochberg et al., 2006, 2012; Santhanam et al., 2006; Moritz et al., 2008; Velliste et al., 2008; Ganguly and Carmena, 2009; Pohlmeyer et al., 2009; Ethier et al., 2012; Collinger et al., 2013a; Wodlinger et al., 2015), brain surface electrodes or electrocorticography (ECoG; Leuthardt et al., 2004; Schalk et al., 2008; Acharya et al., 2010; Chao et al., 2010; Miller et al., 2010; Moran, 2010; Schalk and Leuthardt, 2011; Yanagisawa et al., 2012; Rouse et al., 2013; Wang et al., 2013), electroencephalography (EEG; Wolpaw and McFarland, 2004; Daly and Wolpaw, 2008; Bradberry et al., 2010; McFarland et al., 2010; Doud et al., 2011; Foldes and Taylor, 2011; Ramos-Murguialday et al., 2013), and magnetoencephalography (MEG; Mellinger et al., 2007; Buch et al., 2008; Waldert et al., 2008; Wang et al., 2010b; Sudre et al., 2011; Boe et al., 2014; Florin et al., 2014).

**Figure 1:**
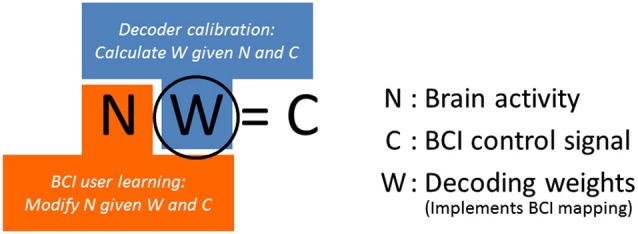
**Decoders can be either linear or non-linear**. For simplicity, we have shown a schematic illustrating the relationship between brain activity (N), BCI control signals (C), and decoding weights (W) for a linear decoder. W implements BCI mapping, i.e., it maps brain activity, N, to a BCI control signal, C.

The central component of a BCI system is its neural decoder, a set of decoding weights that transform or map brain activity to behavior of an external device, e.g., robotic arm movement (Figure 1). Establishment of an effective BCI mapping relies on two synergistic processes (Figure 1). The first is decoder calibration, where decoding weights are calculated based on brain activity and corresponding external device behavior data. The second process is BCI learning, where a BCI user learns the relationship between brain activity and resulting external device behavior given specific decoding weights. In another word, the user learns to generate specific cortical activity patterns for controlling external devices with the given decoding weights.

Researchers have developed many advanced signal processing and neural decoding algorithms for the decoder calibration process (Brockwell et al., 2004; Blankertz et al., 2006; Müller et al., 2008; Yu et al., 2009). In contrast, the BCI learning process is much less understood. To address this knowledge gap, this article will focus on BCI learning with two goals. First, in agreement with recent studies (Yin et al., 2009; Koralek et al., 2012; Rouse et al., 2013; Wander et al., 2013; Sadtler et al., 2014), we contend that BCI learning is analogous to motor and cognitive skill learning and that theories and practice developed for skill learning should inform research in BCI learning. Second, we review approaches that can promote BCI learning, particularly in the context of restoring volitional arm movement or controlling movement of external devices, such as computer cursors and robotic arms. BCI learning is a broad topic and it will be challenging to cover all aspects of BCI learning with reasonable depth in this review article. Hence, this review article focuses on BCI systems that use implantable electrodes, such as ECoG and intracortical microelectrode arrays, with the goal of restoring motor function.

## Types of BCI Mapping

This section discusses BCI mapping in relation to the concept of “mapping” in the field of human-computer interfaces (HCI). BCI systems can be considered as a type of HCI, and BCI research should benefit from established HCI theoretical frameworks. Specifically, the term “mapping” has been widely used in HCI. Norman, a pioneer in HCI research (Norman, 1988), defined mapping as the relationship between human input (e.g., a computer mouse movement) and the resulting behavior of the system under control (e.g., a computer). Analogously, we define BCI mapping as the relationship between brain activity and the resulting behavior of an external device, such as movement of a computer cursor or a robotic arm. BCI mapping can be classified into two main types: biomimetic and artificial. The biomimetic mapping-based BCIs use decoders that aim to capture the natural relationship between cortical activity and volitional arm or hand movement which is then used to control a prosthetic arm, orthosis, or functional electrical stimulator (Georgopoulos et al., 1986; Salinas and Abbott, 1994; Moran and Schwartz, 1999; Brockwell et al., 2004; Paninski et al., 2004; Heldman et al., 2006; Schalk et al., 2007; Wang et al., 2007, 2010a; Shimoda et al., 2012; Chen et al., 2013). This type of mapping potentially provides an intuitive control scheme without undue cognitive load, especially during the initial phase of BCI learning (Wessberg and Nicolelis, 2004). Biomimetic mapping was used to achieve cortical control of high-performance prosthetic limbs using single/multi-unit activities recorded with intracortical microelectrode arrays in individuals with paralysis (Hochberg et al., 2012; Collinger et al., 2013a; Wodlinger et al., 2015).

**Figure 2:**
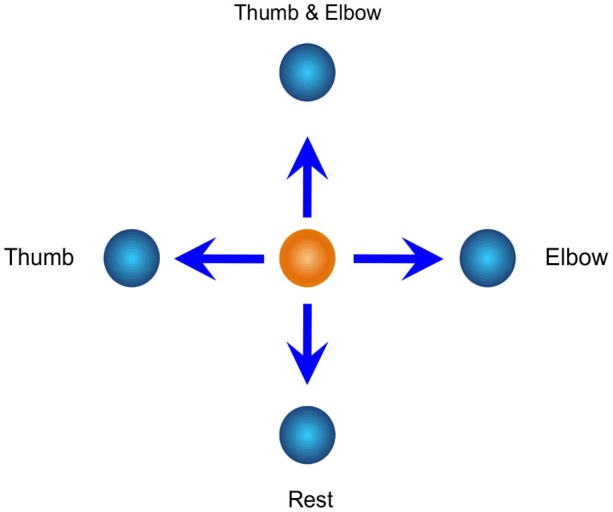
**Artificial mapping for ECoG-based brain control used in Wang et al. (2013)**. Brain activities corresponding to thumb and elbow movements are mapped on to a two-dimensional workspace to serve as the basis for 2D cursor control.

Artificial mapping does not follow the natural relationship between cortical activity and arm/hand movement. Rather, this method either remaps cortical activity into a different movement of a device, or maps cortical activity to device movement using arbitrary decoding weights (Fetz, 2007; Moritz et al., 2008; Schalk et al., 2008; Ganguly and Carmena, 2009; McFarland et al., 2010; Wang et al., 2013). A BCI user has to learn this novel mapping in order to control an external device with his brain activity. For example, Wang et al. remapped cortical activity associated with thumb and elbow movements to two-dimensional (2D) movements of a computer cursor (Figure 2; Wang et al., 2013). During BCI training, the participant was told to associate four flexion/extension movement patterns with four cursor movement directions in *x* — *y* planes. Attempted movements of thumb, elbow, both thumb and elbow, and no thumb or elbow (rest) were associated with for left, right, up and down, respectively. It is also worth noting that this approach allowed the participant to move the cursor in any directions in the 2D workspace and not just up, down, left, and right. The participant, an individual with long-term paralysis due to cervical spinal cord injury, learned this mapping and achieved cortical control of a computer cursor.

There is no strict division between biomimetic and artificial BCI mapping; rather both are parts of a continuous spectrum. Most biomimetic mapping-based studies use neural recording technologies that provide a small and potentially noisy and biased sample of the total neuronal population that controls natural limb movement. Thus, even though biomimetic mapping is intended to be intuitive, BCI users likely have to undergo a certain degree of BCI learning. Conversely, artificial mapping-based studies often build BCI learning on top of existing cortical activity patterns that naturally represent certain neuronal processes, such as those for mouth and limb movement control (Schalk et al., 2008; McFarland et al., 2010; Wang et al., 2013).

## Establishing the Initial BCI Mapping Using Movement-Related Paradigms

Before any BCI learning can take place, we first need to establish the initial mapping between brain activity and intended device behavior. This is the neural decoder calibration process. While it is possible to just use a set of arbitrary or random decoding weights (Ganguly and Carmena, 2009), the typical practice is to calculate decoding weights using neural data and corresponding limb movement data. This practice is motivated by previous neurophysiology studies which have demonstrated that neurons in various cortical areas, most notably in the motor and premotor areas, fire in specific and reliable ways during the execution of volitional movements (Georgopoulos et al., 1986; Moran and Schwartz, 1999; Paninski et al., 2004; Wang et al., 2007, 2010a; Truccolo et al., 2008; Kaufman et al., 2010). A common approach for decoder calibration for individuals who are able to move their arm is the following: first, have the subject perform a series of overt movements; second, calculate decoding weights with a certain algorithm, such as the population vector algorithm (Georgopoulos et al., 1986), or the optimal linear estimator (Salinas and Abbott, 1994), and the decoding weights capture the relationship between brain activity and natural arm movement; last, the decoding weights map brain activity to real-time control signals for external devices (Helms Tillery et al., 2003).

Clinical BCI users typically have limb paralysis or dysfunction, thus making it difficult or impossible to use overt movements for decoder calibration. One way to address this problem is to derive decoding weights using action observation paradigms based on the concept of the mirror neuron system (MNS). The MNS is a collection of neurons in the premotor and inferior parietal areas that fire both when an individual acts and when the individual observes the same action performed by another person (Buccino et al., 2004; Iacoboni and Dapretto, 2006; Rizzolatti and Sinigaglia, 2007; Fabbri-Destro and Rizzolatti, 2008). Studies in non-human primates (Tkach et al., 2007, 2008; Velliste et al., 2008) and able-bodied human subjects (Hari et al., 1998; Iacoboni and Dapretto, 2006; Perry and Bentin, 2009; Press et al., 2011; Collinger et al., 2014) suggest that primary motor cortex also demonstrates congruent activities during both action observation and action execution. Reliable motor cortical activation during action observation was also observed in individuals with chronic limb paralysis (Dushanova and Donoghue, 2010; Wang et al., 2013; Collinger et al., 2014). Therefore, action observation can be an effective approach for identifying the mapping between motor cortical activity and limb movement in absence of overt movement.

Another approach for neural decoder calibration in individuals with limb paralysis is to use motor imagery, which activates a cortical substrate similar to that of overt movement (Porro et al., 1996; Crammond, 1997; Jeannerod and Frak, 1999; McFarland et al., 2000; Jeannerod, 2001; Sharma et al., 2006). Research has demonstrated that able-bodied individuals can perform motor imagery to successfully operate BCI systems (Pfurtscheller and Neuper, 2001; Leuthardt et al., 2004; Schalk et al., 2008; Blankertz et al., 2010; Miller et al., 2010). In addition to action observation and motor imagery, simply instructing individuals with paralysis to attempt limb movement can reliably activate the motor and somatosensory cortices. BCI studies in individuals with paralysis have used this approach for both decoder calibration and brain control (Hochberg et al., 2006, 2012; Truccolo et al., 2008; Collinger et al., 2013a; Wang et al., 2013). Combination of the above techniques, such as action observation and attempted movement, will likely yield strong and reliable activation of the motor cortical areas for the initial neural decoder calibration in individuals with limb paralysis.

## BCI Learning and Motor/Cognitive Skill Learning

BCI learning and motor/cognitive skill learning share many common characteristics, including the learning stages and neural substrates that support learning. In terms of learning stages, Fitts and colleagues suggested that motor/cognitive learning follows three stages (Fitts and Posner, 1967; VanLehn, 1996): (1) cognitive stage—an individual learns basic information about the goals and parameters of the task, i.e., learns what to do; (2) associative stage—an individual learns to convert their knowledge about the task into actual action, i.e., learns how to do; (3) automatic stage—an individual performs a task automatically, with minimal effort, and independent of conscious awareness (Logan, 1988). The automatic process requires little or no conscious effort, particularly in terms of working memory and attention. This process reduces mental fatigue, and enables an individual to multitask. BCI learning seems to go through similar stages. Human subjects of BCI studies have anecdotally reported transitioning from a very deliberate cognitive stage to a nearly automatic stage after practice (Curran and Stokes, 2003; Wander et al., 2013). The ability and time for a BCI user to arrive at the automatic stage will likely vary, depending on neural recording modalities, BCI mapping strategies, complexity of devices to be controlled, a user’s cognitive functions, etc. Nevertheless, once learning reaches the automatic stage, a BCI user should be able to multitask, such as carry on a conversation with someone else while controlling a cursor using brain activities (Miner et al., 1998; Foldes and Taylor, 2013).

In terms of neural substrates, during BCI learning, there is significant involvement of a distributed network spanning the motor cortex, prefrontal area, parietal area, cerebellum, and striatum, all of which are also engaged during motor/cognitive skill learning (Doyon and Benali, 2005; Wander et al., 2013). Part of this network’s activity decreases during BCI learning as a subject transitions from the cognitive to automatic stage (Wander et al., 2013). Furthermore, plasticity in corticostriatal circuits has been implicated in motor learning (Barnes et al., 2005; Kimchi and Laubach, 2009; Yin et al., 2009), and corticostriatal plasticity seems to be necessary for BCI learning, as well (Koralek et al., 2012). Overall, BCI learning seems to capitalize on many of the same neural circuitries involved in motor and cognitive skill learning.

Another important aspect of BCI learning to consider is “BCI illiteracy”, which has been observed in EEG studies (Vidaurre and Blankertz, 2010). BCI illiteracy describes the phenomenon that certain individuals had much more difficulty in learning BCI control than others. One study reported that an estimated 15–30% of the study participants did not achieve proficient BCI control by the end of the study (Dickhaus et al., 2009). Poor BCI performance was attributed to individuals using a wrong strategy of imagining a movement instead of imaging the kinesthetic movement, and reduced modulation depth of sensorimotor rhythm during motor imagery for EEG (Blankertz et al., 2009). BCI illiteracy has not been reported by BCI studies using implantable electrodes, but the number of subjects studied is typically small (Leuthardt et al., 2004; Hochberg et al., 2012; Collinger et al., 2013a; Wang et al., 2013). Further studies are needed to better understand the underlying cause of BCI illiteracy and identify new BCI learning strategies that will alleviate BCI illiteracy.

## Approaches for BCI Learning

During BCI learning, an individual learns to generate specific cortical activity patterns to control external devices. Similar to motor/cognitive skill learning, BCI learning produces neural adaptation in the form of functional reorganization of the cortex and changes in neuronal tuning properties (Taylor et al., 2002; Carmena et al., 2003; Neumann et al., 2004; Jarosiewicz et al., 2008; Chase et al., 2012). The duration of time required for BCI learning is associated with BCI user performance, which in turn is related to the paradigm and neural recording modality used in the study. Schalk et al. showed that five human subjects used ECoG to achieve 2D cursor control within a single session (training period of 12–36 min; Schalk et al., 2008). Wang et al. showed that a participant with tetraplegia was able to perform 3D cursor control using ECoG signals in 6 days of BCI training, with training sessions lasting between 4–6 h (Wang et al., 2013). Collinger et al. tested an intracortical microelectrode-based BCI system in an individual with tetraplegia (Collinger et al., 2013a). They reported that 3D control was achievable within a single session of BCI training and that 7D control of a robotic arm was achieved after 66 days of BCI training, with BCI training taking place three times a week and for about 3 h each time. Given the essential role of learning in any BCI application, it is important to survey and understand effective approaches that can promote BCI learning. These approaches can be roughly classified into five types: computer-assisted learning, co-adaptive learning, operant conditioning, sensory feedback, and cortical stimulation. These approaches are not mutually exclusive, and researchers often combine some of these approaches to facilitate BCI learning.

### Computer-Assisted Learning

**Figure 3:**
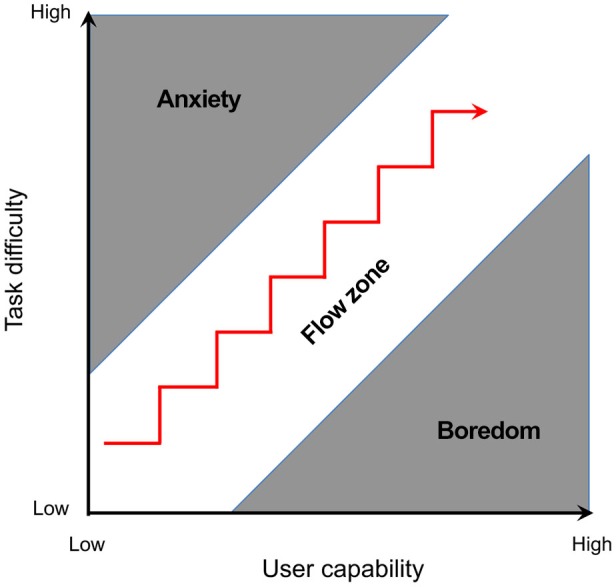
**Graph of Csikszentmihalyi’s flow state when a person’s ability to execute a task balances the difficulty of the task they have to perform (Figure adapted from Csikszentmihalyi, 1990)**.

During the initial stage of BCI learning, researchers often use computer assist to help users learn to modulate brain signals to control external devices. The process of computer-assisted learning can be discussed using two concepts from the psychology of learning. The first is the concept of “flow zone”, which was introduced by Csikszentmihalyi and widely used in game design (Figure 3; Csikszentmihalyi, 1990; Dickey, 2007; Schell, 2008; Christel et al., 2013). In the flow zone, the task difficulty is balanced against a person’s capability to keep the individual engaged with the learning process without stress or boredom. The second concept is “shaping”, originally proposed by Skinner (Ferster and Skinner, 1957; Skinner and Ferster, 1997; Gluck et al., 2008). Shaping describes a successive approximation process during which the task goal is morphed gradually from coarse to fine, in order to help an individual refine performance and eventually perform a complex task with high precision. For example, learning how to play tennis, an individual will first learn to hit the ball across the court, and then gradually learn to control where the ball lands. The concepts of flow zone and shaping are highly connected to each other. As the task difficulty increases, the goal becomes increasingly complex and specific. By regulating task difficulty, shaping helps keep an individual in the flow zone throughout the learning process.

For BCI learning, computer assist is a powerful means to control task difficulty and keep the subject in the flow zone while shaping brain control performance. Summarizing previous studies (Taylor et al., 2002; Velliste et al., 2008; Collinger et al., 2013a,b; Rouse et al., 2013; Wang et al., 2013), computer assist is the approach where the control signal derived from brain activity is combined with an assistive signal automatically generated by a computer to help the subject learn brain control of devices. Here we describe computer assist in the context of cursor control for convenience of discussion. Generally speaking, there are two types of computer assists: active assist and passive assist (Velliste et al., 2008; Collinger et al., 2013a; Wang et al., 2013). Active assist directly drives the cursor toward the target location, whereas passive assist only constrains the deviation of cursor movement from the ideal trajectory without actively pushing the cursor toward the target location. Helms Tillery et al. have indicated that computer-assisted BCI learning led to greater learning rates than BCI learning without any assist (Helms Tillery et al., 2003). Furthermore, an effective training schedule to adjust the level of computer assist and task difficulty is critical to keep the subject in the flow zone and thus promote rapid BCI learning. While such adjustments are often done empirically, Zhang et al. has proposed an adaptive algorithm to automatically adjust computer assist level based on subject performance in order to maximize the learning rate (Zhang et al., 2012). Lastly, an important factor to consider is that computer assist can mask or distort the actual effect of brain control signals and impair BCI learning. In our experience, when the amplitude of computer assist is comparable to the brain control signal the resulting cursor movement can confuse the subject and even hinder the learning process. More rigorous modeling and experimental studies are needed to better inform the field about what type of computer assist to use, when to use or adjust computer assist, and how much computer assist should be applied to maximize the learning rate.

### Co-Adaptive BCI Learning

BCI learning has the potential to induce cortical plasticity, manifested as changes in both modulation depth and preferred directions of neurons used for BCI control (Taylor et al., 2002; Jarosiewicz et al., 2008). To maximally capitalize on these cortical changes, researchers proposed the concept of “co-adaptation”, which involves both periodic calibration of neural decoder and the brain adapting to the neural decoder (Taylor et al., 2002; Sanchez et al., 2009). One specific form of co-adaptive learning is the “turn-taking adaptation” method used in our previous study, where the subject and neural decoder took turns to adapt to each other (Wang et al., 2013). During the user learning period, the neural decoder remained constant for 5 blocks of 20 trials of real-time BCI operation (approximately 15 min). During the decoder calibration period, the subject was instructed to behave consistently for 5 blocks of 20 trials (15 min), i.e., use the same attempted movement scheme, to generate consistent neural data for decoder calibration. The decoder was recalibrated either daily or when the subject’s performance plateaued. Another approach used by our study was “incremental learning”, where each BCI session always started with the neural decoder used at the end of the previous session (Wang et al., 2013). This approach was possible given the stability of the ECoG signals. Differing from previous approaches where a new decoder was calculated at the beginning of each day’s testing, the incremental learning approach enabled the subject to build upon what he learned from previous sessions (Ganguly and Carmena, 2009).

### Operant Conditioning

Operant conditioning is a learning process that makes subjects associate a particular behavior with a specific consequence through reinforcements. The reinforcement is provided when a subject completes a trial successfully, and it can be juice or food rewards in animal studies, and provision of a token/award or increments in scores in human studies. In the field of BCI, operant conditioning is typically used to train experimental animals to discover the underlying BCI mapping by trial and error, without explicit instructions (Chase and Schwartz, 2011; Arduin et al., 2013). Operant conditioning of brain activity through biofeedback is a particularly useful paradigm for learning arbitrary BCI mappings (Fetz, 1969, 2007; Ganguly and Carmena, 2010; Engelhard et al., 2013). In this approach subjects learn to generate specific brain activity patterns based on real-time sensory feedback (visual, auditory, tactile, etc.) to complete a BCI task. Operant conditioning can modulate single neuron firing rates (Fetz, 1969), ensemble neuronal activity (Ganguly and Carmena, 2009), neuronal synchrony (Engelhard et al., 2013), and high gamma band power of field potential signals (Rouse et al., 2013; Wander et al., 2013). Through operant conditioning an ensemble of neurons can potentially assume a novel yet reproducible pattern of activity, allowing the subject to achieve reliable brain control of a device.

Interestingly, through operant conditioning subjects sometimes acquire brain control of an external device without being consciously aware of how they are performing the task (Kaplan et al., 2005). This is similar to implicit learning (Frensch and Rünger, 2003), which is defined as “non-episodic learning of complex information in an incidental manner, without awareness of what has been learned” (Seger, 1994). In other words, individuals learn certain skills without being aware that learning has occurred (Gluck et al., 2008). Implicit learning in the context of BCI learning is a scientifically intriguing and clinically relevant topic to investigate.

### Sensory Feedback

Many approaches discussed so far make substantial use of sensory feedback. Feedback is an essential component of BCI learning, therefore we are devoting this section for sensory feedback. Borrowing concepts from motor learning, we will discuss two types of feedback used in BCI learning: the online feedback that provides detailed knowledge of performance (KP) and the offline feedback that provides the knowledge of results (KR), i.e., success or failure with respect to the goal (Schmidt, 2008). Both KP and KR feedbacks are crucial for effective BCI learning. KP feedback provides continuous or frequent feedback to help the participant complete individual trials. For example, in a typical cursor control task, KP feedback is provided to a user visually as movement of the cursor under brain control. KR feedback is provided at the end of a trial or a session, often through either auditory tones indicating success or failure of a trial or simply feedback about success rates.

Feedback can also be categorized based on the sensory modality it uses, such as visual, auditory, and somatosensory feedback. Research has demonstrated that visual feedback plays a key role in skill learning (Hinterberger et al., 2004; Abbott, 2006; Leeb et al., 2006; Blankertz et al., 2008; Barbero and Grosse-Wentrup, 2010). In addition to visual feedback, the BCI field has seen a significant increase in the use of somatosensory feedback to further improve BCI performance. Loss of somatosensory feedback significantly impairs motor performance, particularly for grasping and object manipulation (Macefield et al., 1996; Monzée et al., 2003; Goodwin and Wheat, 2004; Bensmaia and Miller, 2014). Lack of somatosensory information may also result in poor motor planning (Brochier et al., 1999). Thus, it is critical for a BCI system to have the capability to provide somatosensory feedback, especially for brain control of prosthetic arms. A number of BCI studies have demonstrated that electrical stimulation of the peripheral nerve, dorsal root ganglia, or the somatosensory cortex can elicit artificial sensation that the subjects can use to perform sensory discrimination tasks or even closed-loop brain-control tasks successfully (Romo et al., 2000; Horch et al., 2011; Miller and Weber, 2011; O’Doherty et al., 2011; Weber et al., 2011, 2012; Johnson et al., 2013; Tabot et al., 2013; Zaaimi et al., 2013; Bensmaia and Miller, 2014).

### Cortical Stimulation

Since cortical stimulation can modulate cortical activity patterns (Hummel and Cohen, 2006; Harvey and Nudo, 2007), it is conceivable that cortical stimulation may be able to replace or supplement repetitive behavior training to induce changes in cortical activity and accelerate BCI learning (Soekadar et al., 2014). While this approach has not been well investigated for BCI learning, previous studies about neuroplasticity (Gage et al., 2005; Jackson et al., 2006) and rehabilitation using neurostimulation (Ziemann et al., 2002; Hummel et al., 2005; Hummel and Cohen, 2006; Harvey and Nudo, 2007; Perez and Cohen, 2009; Plow et al., 2009; Reis et al., 2009) can shed some light on the feasibility of this approach. At the macroscopic level, cortical areas can be stimulated non-invasively using transcranial magnetic or current stimulations. In the context of stroke rehabilitation, it has been suggested that such stimulation can enhance motor cortical excitability and change cortical connectivity (Hummel et al., 2005; Hummel and Cohen, 2006; Perez and Cohen, 2009). At the microscopic level, based on the concept of Hebbian or associative learning, motor cortical reorganization can be induced by coupling action potentials of one motor cortical neuron with electrical stimulation impulses of another motor cortical neuron (Jackson et al., 2006; Stevenson et al., 2012). A recent pilot study has shown that transcranial direct current stimulation induces event-related desynchronization associated with sensorimotor rhythm (Wei et al., 2013). This event-related desynchronization, along with motor imagery, was used to improve the performance of an EEG based BCI. Besides electromagnetic stimulation, optogenetics is another approach to stimulate cortical tissue. This technique uses light to activate neurons that have been genetically modified to have light-sensitive ion channels. Optogenetics has enabled manipulation of neuronal activity with much higher spatial and temporal precisions than was previously possible (Tye and Deisseroth, 2012). Lima and Miesenbock demonstrated reliable control of neuronal spiking in the millisecond-timescale and control of excitatory and inhibitory synaptic transmission (Lima and Miesenböck, 2005). Optogenetics is currently limited to animal research as it requires genetic manipulation (Chow and Boyden, 2013), but this technique has great potential for facilitating learning by inducing repeatable patterns of neural activity.

Given all these possibilities of directly modulating cortical activity and connectivity, we believe that cortical stimulation can be a powerful approach to promote BCI learning. Cortical stimulation may not only be able to modify general cortical excitability at a macroscopic level, but also directly entrain cortical activity into specific spatiotemporal patterns for effective brain control of external devices.

## Conclusion

In this article, we provided an overview of BCI learning by discussing BCI mapping, relationship between BCI learning and motor/cognitive skill learning, and approaches for accelerating BCI learning. We believe that advancement in theories and practice of BCI learning, coupled with development of clinically reliable neural interfaces, will ultimately benefit many individuals with disabilities and our society.

## Conflict of Interest Statement

The Guest Associate Editor Martin Oudega declares that, despite being affiliated to the same institution as the authors, the review process was handled objectively and no conflict of interest exists. The authors declare that the research was conducted in the absence of any commercial or financial relationships that could be construed as a potential conflict of interest.
